# Analysis of resistance genes of carbapenem-resistant *Providencia rettgeri* using whole genome sequencing

**DOI:** 10.1186/s12866-023-03032-3

**Published:** 2023-10-03

**Authors:** Mi Liu, Na Yi, Xinyi Wang, Rongrong Wang

**Affiliations:** 1 Department of Clinical Laboratory, Weifang People’s Hospital, Weifang, Shandong China; 2 Clinical Medicine, Shandong First Medical University, Taian, Shandong China

**Keywords:** Whole genome sequencing, Carbapenem-resistant *Providencia rettgeri*

## Abstract

**Objective:**

This study aimed to investigate the clinical infection characteristics and analyze the resistance gene carrying status of carbapenem-resistant *Providencia rettgeri* via whole genome sequencing (WGS).

**Methods:**

Carbapenem-resistant *P. rettgeri* were collected from clinical patients between January 2020 and December 2021, and their susceptibility to 19 antimicrobial drugs was determined using the VITEK 2 Compact system and Kirby–Bauer (KB) disk diffusion method. The Illumina platform was used to perform WGS of the *P. rettgeri* isolates, and the resistance genes carried by the Carbapenem-resistant *P. rettgeri* strains were detected via ABRicate software. The phylogenetic tree was constructed by thirty-four strains including twenty-eight strains downloaded from NCBI database and the carbapenem-resistant six *P. rettgeri* strains in this study. Which based on genomic single nucleotide polymorphism (SNP) to understand the affinities of the carbapenem-resistant *P. rettgeri* strains.

**Results:**

Six carbapenem-resistant *P. rettgeri* strains were isolated from five different clinical departments using the blood, urine, sputum, and secretion specimens. These infected patients are middle-aged and elderly people with a history of severe trauma, tumors, hypertension, and various other underlying diseases, and invasive procedures. Antimicrobial sensitivity testing showed that all strains presented resistance to ampicillin-sulbactam, ceftazidime, ciprofloxacin, levofloxacin, and ertapenem, whereas they exhibited full susceptibility to cefepime and amikacin. Most strains demonstrated high resistance to β-lactams, aminoglycosides, and sulfonamides. Thirty-five resistance genes were identified by ABRicate. All carbapenem-resistant *P. rettgeri* strains carried aminoglycoside, fluoroquinolone, chloramphenicol, rifampicin, sulfonamide, and β-lactam resistance genes, and most importantly, all strains possessed the carbapenem resistance gene *bla*_NDM−1_. The six *P. rettgeri* strains in this study and the 28 carbapenem-resistant *P. rettgeri* strains from the NCBI database were divided into four evolutionary groups. The WF3643, WF3849, WF3822, and WF3821 strains in this study were in the same evolutionary group (clade A), while the closely related WF3099 and WF3279 strains were in different evolutionary groups (clade B and clade D), respectively. The WF3099 strain was distantly related to the other five strains.

**Conclusion:**

Carbapenem-resistant *P. rettgeri* strains were mostly isolated from middle-aged and older patients with a history of surgery or serious underlying diseases, and they were found to cause multisystem infections. All Carbapenem-resistant *P. rettgeri* strains in this study carried *bla*_NDM−1_ and multiple antimicrobial drug resistance genes. Furthermore, the *P. rettgeri* strains in this study were closely related, suggesting the possibility of nosocomial infections. Therefore, our study highlights the need for research on *P. rettgeri* to control the spread of these nosocomial infections.

**Supplementary Information:**

The online version contains supplementary material available at 10.1186/s12866-023-03032-3.

## Introduction

*Providencia* spp. are a group of urease-producing gram-negative bacteria belonging to the *Enterobacteriaceae* family, including *P. rettgeri*, *P. stuartii*, *P. alcalifaciens*, *P. heimbachae*, and *P. rustigianii* [1]. These bacteria are part of the normal intestinal flora; however, under certain conditions, they can not only cause urinary tract infections and diarrhea but also serious infectious diseases such as pneumonia, endocarditis, sepsis, and meningitis. Moreover, the *Providencia* genus is an important conditional pathogen of hospital-acquired infections [2, 3].

The unreasonable and irregular application of antimicrobial drugs, coupled with the preference and increased dosage of carbapenem antimicrobial drugs in recent years has increased the severity of multi-resistant and pan-resistant bacteria. The emergence of carbapenem⁃resistant *Enterobacteriaceae* poses a serious threat to human health. Carbapenems are the most potent β-lactams used for the treatment of gram-negative bacillary infections, particularly those caused by *Enterobacteriaceae*. NDM-1 is a class B β-lactamase encoded by the *bla*_NDM−1_ gene and confers resistance against all β-lactam antibiotics, including carbapenems [4]. After the 2010 report on *bla*_NDM−1_, its corresponding enzyme has received widespread global attention, with outbreaks of NMD-1-producing bacteria being reported in several countries and regions, including Hong Kong and mainland China.

In recent years, the occurrence of *P. rettgeri* infections along with an increased detection rate of multi-drug resistant bacteria has been reported. In particular, carbapenem-resistant *P. rettgeri* has been emerging as one of the pathogens causing hospital infections, posing serious challenges to clinical disease diagnosis and treatment [5]. In this study, we aimed to analyze the drug resistance of carbapenem-resistant strains of *P. rettgeri* in our hospital using their whole genome sequencing data and elucidate their drug resistance gene carriage and homologous evolutionary relationship of the nosocomial strains. This study will provide useful data for the treatment selection of clinical antimicrobial drugs as well as for the prevention and control of hospital infections.

## Materials and methods

### Collection of clinical isolates

Between January 2020 and December 2021, six carbapenem-resistant *P. rettgeri* strains were isolated from patients visiting a 3,000-bed-capacity tertiary teaching hospital in northern China. The bacterial strains were identified using matrix-assisted laser desorption/ionization time-of-flight mass spectrometry (MALDI-TOF-MS) via the VITEK MS system (Sysmex-bioMerieux, Marcy l’Etoile, France) and average-nucleotide identity analysis based on whole genome sequence data.

### Antimicrobial susceptibility testing

Antimicrobials commonly used in the clinic include piperacillin-tazobactam(TZP), gentamicin(GEN), cefepime (FEP), imipenem(IPM), ceftriaxone(CRO), tobramycin(TOB), amikacin (AMK), ceftazidime (CAZ), ertapenem (ETP), levofloxacin (LVX), trimethoprim-sulfamethoxazole (SXT), ampicillin-sulbactam (SAM), and ciprofloxacin (CIP) was performed using the VITEK 2 Compact (GN13) system. Antimicrobial susceptibility testing for the drugs not included in the GN13 card, including cefoxitin(FOX), cefuroxime(CXM), aztreonam(ATM), meropenem(MEM), cefoperazone/sulbactam(CSL), and cefotaxime (CTX) was conducted using the Kirby–Bauer (KB) disk diffusion method (Oxoid, Hampshire, United Kingdom). The susceptibility testing results were interpreted according to the 2022 Clinical and Laboratory Standards Institute guidelines (https://clsi.org) [6]. *Escherichia coli* ATCC 25,922 and *pseudomonas aeruginosa* ATCC27853 were used as the quality control strain.

### Whole genome sequencing and assembly

Bacterial DNA was extracted using Omega Bio-Tek Bacterial DNA Kit (Doraville, GA, USA) after overnight culture of the strains at 37 °C. DNA library preparation was performed either using the Illumina Nextera XT DNA library preparation kit (Illumina, USA) according to the manufacturer’s instructions prior to sequencing on Illumina HiSeq or MiSeq platform. Raw sequences were trimmed for quality, followed by assembly and annotation. Low-quality reads and adapters were trimmed using TrimGalore (v0.4.5, https://github.com/FelixKrueger/TrimGalore) and assembled via SPAdes (v3.13.0) (http://bioinf.spbau.ru/spades) using default parameters [7].

### Whole genome sequencing analysis

The NCBI AMRFinder database was used to detect antimicrobial resistance genes using ABRicate (v0.9.8, https://github.com/tseemann/abricate) [8]. Plasmids were searched using PlasmidFinder [9]. Except for the data of the six strains in this study, the data of the other carbapenem-resistant *P. rettgeri* strains required for constructing the phylogenetic tree were downloaded from the NCBI database. Consequently, the complete genomic data of 28 carbapenem-resistant *P. rettgeri* strains were obtained from the NCBI database (up to January 31, 2023), which were then identified to contain carbapenem resistance genes (*bla*_NDM−1_) by ABRicate (Table S1). Based on the core genomes of the *Providencia* spp., phylogeny was used for single nucleotide polymorphism(SNP) analysis. MEGAX 10.1.8 was used to generate unrooted maximum-likelihood phylogenetic trees with a bootstrap iteration of 1000 [10]. The phylogenetic tree was visualized by using iTOL [11].

## Results

**Characteristics of the patients with carbapenem-resistant*P. rettgeri***.

**Fig. 1:**
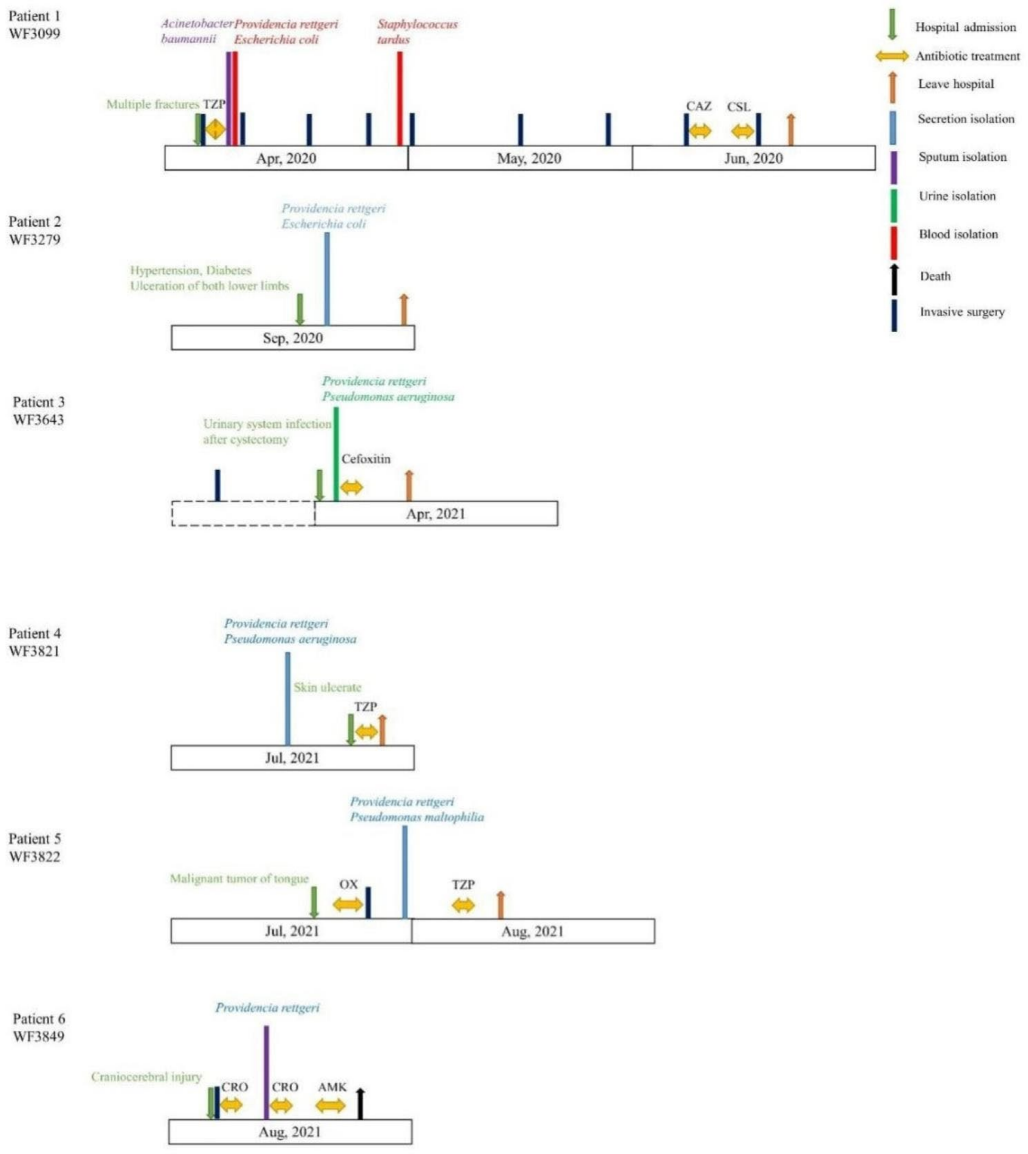
Carbapenem-resistant *P. rettgeri* infectious cases

Among the six patients in this study, four patients were male and two were female, with an age range of 21–69 years and mean age of 51 years (Table S2 and Fig. 1).

Patient 1 (WF3099) was in the intensive care unit (ICU) with multiple fractures. The patient was hospitalized in the ICU for 76 days and had undergone several invasive procedures, including subcutaneous tissue debridement and internal fixation of pelvic fractures. Patient 1 was discharged in good condition.

Patient 2 (WF3279) was in the cardiology department with severe underlying disease that resulted in bilateral lower extremity ulceration. The patient’s secretions were cultured for *P. rettgeri*. Patient 2 was discharged in fair condition, with unremitting bilateral lower extremity ulceration.

Patient 3 (WF3643) had undergone cystectomy for a urologic malignancy and was hospitalized due to a urinary tract infection. *P. rettgeri* was isolated from the patient’s urine specimen. Patient 3 was treated and discharged in good condition.

Patient 4 (WF3821) was an outpatient who had skin ulceration for 4 months. *P. rettgeri* was cultured from the patient’s secretions. Patient 4 was hospitalized for 1 day and was discharged in poor condition with no follow-up records.

Patient 5 (WF3822) had a malignant tumor of the tongue. After tongue resection, *P. rettgeri* was isolated from the patient’s secretions. Patient 5 was discharged in good condition.

Patient 6 (WF3849) had undergone craniocerebral hematoma removal for craniocerebral trauma. *P. rettgeri* was isolated from the patient’s sputum. Patient 6 eventually died.

**Antimicrobial sensitivity characteristics of carbapenem-resistant*P. rettgeri***.

Six *P. rettgeri* strains showed multi-antibiotic resistance (Table 1), among which five strains were resistant to more than 10 antibiotics and one strain was resistant to eight antibiotics. All strains showed resistance to SAM, CAZ, CIP, LVX, and ETP, whereas they demonstrated full susceptibility to FEP and AMK.

**Table 1:** Antimicrobial sensitivity of carbapenem-resistant *P. rettgeri*

	Antimicrobial drugs	WF3099	WF3279	WF3643	WF3821	WF3822	WF3849
β-lactam/β-lactamase inhibitor combination	SAM	R	R	R	R	R	R
	TZP	R	S	S	S	S	R
	CSL	R	I	R	S	I	R
	CAZ	R	R	R	R	R	R
	CRO	I	R	R	R	R	R
	FEP	S	S	S	S	S	S
	CTX	I	I	R	R	R	R
	CXM	I	R	R	R	R	R
	FOX	S	S	I	S	I	I
	ATM	S	S	S	S	S	R
	IPM	I	R	R	R	R	R
	ETP	R	R	R	R	R	R
	MEM	S	R	S	R	R	R
Aminoglycosides	GEN	I	R	R	I	R	S
	TOB	I	R	R	S	I	S
	AMK	S	S	S	S	S	S
Fluoroquinolones	CIP	R	R	R	R	R	R
	LVX	R	R	R	R	R	R
Sulfonamides	SXT	R	R	R	S	R	S

*ceftriaxone (CRO), amikacin (AMK), gentamicin (GEN), aztreonam (ATM), ceftazidime (CAZ), ciprofloxacin (CIP), levofloxacin (LVX), cefepime (FEP), imipenem (IPM), ertapenem (ETP), trimethoprim-sulfamethoxazole (SXT), tobramycin (TOB), piperacillin-tazobactam (TZP), ampicillin-sulbactam (SAM), meropenem (MEM),cefuroxime (CXM), cefotaxime (CTX), cefoperazone/sulbactam (CSL) and cefoxitin (FOX)

The six *P. rettgeri* strains contained 35 drug resistance genes. Among them, the WF3099 strain carried 19 types of resistance genes, WF3279, 23; WF3643, 17; WF3821, 15; WF3822, 15; and WF3849, 9. All strains harbored aminoglycoside, fluoroquinolone, chloramphenicol, rifampicin, sulfonamide, and carbapenem resistance genes (Table 2). Lastly, all six strains possessed the carbapenem resistance gene *bla*_NDM−1_.

**Table 2:** Resistance genes, resistance phenotypes and plasmid analysis of carbapenem-resistant *P. rettgeri*

Strain	Plasmid type	Resistant genes										Phenotype			
		Aminoglycosides	Fluoroquinolones	Macrolides	phenicol	Fosfomycin	Rifampicin	Sulfonamides	tetracycline	trimethoprim	β-lactams	Aminoglycosides	Fluoroquinolones	Sulfonamides	β-lactams
WF3099	/	*aph(4)-Ia* *aac(3)-IV* *aph(3’’)-Ib* *aph(6)-Id* *aph(3’)-Ia* *aph(3’)-VIa* *aadA2*	*qnrA1*	*lnuF* *mphE* *msrE*	*floR*	/	*Arr3*	*sul1* *sul2*	*tet(59)*	*dfrA12*	*bla*_OXA−1_ *bla*_NDM−1_	/	CIP-LVX	SXT	SAM-TZP-CAZ-CSL- ETP
WF3279	repUS18_4_rep(pSA8589) Col3M_1	*aac(3)-IV* *aph(4)-Ia* *aph(3’’)-Ib* *aph(6)-Id* *ANT(2’’)-Ia* *aadA16* *aph (3’)-Ia*	*qnrD1*	*cfrA* *mphE* *msrE*	*catB8* *floR*	/	*Arr3*	*sul1* *sul2* *sul3*	*tet(B)* *tet(59)*	*dfrA27*	*bla*_OXA−1_ *bla*_OXA−10_ *bla*_NDM−1_	GEN-TOB	CIP-LVX	SXT	SAM-CAZ-CRO-CXM- IMP-MEM-ETP
WF3643	Col3M_1	*aph(3’’)-Ib* *aph(6)-Id* *ANT(2’’)-Ia*	*qnrA1* *qnrD1*	*lnuF* *mphE* *msrE* *lnuG*	*catB8*	*FosA3*	*Arr3*	*sul1* *sul2*	*tet(B)*	/	*bla*_OXA−10_ *bla*_NDM−1_	GEN-TOB	CIP-LVX	SXT	SAM-CAZ-CRO-CTX-CXM-CSL- IMP-ETP
WF3821	*/*	*aph(6)-Id* *aph(3’’)-Ib* *aph(3’)-Ia* *ANT(2’’)-Ia* *ANT(3’’)-IIa*	*qnrA1*	*/*	*catA1* *catB8* *floR*	/	*Arr3*	*sul1* *sul2*	*tet(B)*	/	*bla*_OXA−10_ *bla*_NDM−1_	/	CIP-LVX	/	SAM-CAZ-CRO-CTX-CXM-IMP-MEM-ETP
WF3822	Col3M_1	*aph(4)-Ia* *aac(3)-IV* *aph (3’)-Ia* *aadA2*	*qnrD1*	*lnuG* *mphE* *msrE*	*floR*	/	*Arr3*	*sul1* *sul2*	*tet(B)*	*dfrA12*	*bla*_NDM−1_	GEN	CIP-LVX	SXT	SAM-CAZ-CRO-CTX-CXM-IMP-MEM-ETP
WF3849	Col3M_1	*ANT(2’’)-Ia* *ANT(3’’)-IIa*	*qnrD1*	*/*	*cmlA5*	/	*Arr2*	*sul1*		/	*bla*_OXA−10_ *bla*_VEB−1_ *bla*_NDM−1_	/	CIP-LVX	/	SAM-TZP-CAZ-CRO-CTX-CXM-CSL-IMP-MEM-ETP

* The grey part indicates carbapenem resistance genes and carbapenem antibacterial drugs / represents no resistance genes or no resistant antimicrobial drugs or no plasmids

The examination of the resistance genotypes and phenotypes of *P. rettgeri* against four categories of antibiotics, namely aminoglycosides, fluoroquinolones, β-lactam/β-lactamase inhibitor combinations, and sulfonamides, and the results of antimicrobial sensitivity testing revealed that the strains of *P. rettgeri* exhibited a high degree of uniformity in their resistance genotypes and phenotypes. Among them, *P. rettgeri* carrying fluoroquinolone resistance genes (*qnrA1 or qnrD1*) were resistant to CIP and LVX, while those possessing β-lactam resistance genes demonstrated high resistance to β-lactam/β-lactamase inhibitor combination antimicrobials. All six *P. rettgeri* strains harboring *bla*_NDM−1_ showed resistance to carbapenems, consistent with the resistance phenotype. Interestingly, the WF3099, WF3821, and WF3849 strains carried aminoglycoside resistance genes but did not exhibit resistance to aminoglycoside antimicrobials. Similarly, the WF3821 and WF3849 strains possessed sulforaphane resistance genes but demonstrated sensitivity to SXT.

The PlasmidFinder prediction analysis (Table 2) showed that four of the six carbapenem-resistant *P. rettgeri* strains carried plasmids, with the WF3279 strain carrying two plasmids (repUS18_4_rep(pSA8589) and Col3M_1) and the remaining three strains (WF3643, WF3822, and WF3849) harboring one plasmid (Col3M_1). Plasmid repUS18_4_rep(pSA8589) is identical to ACCESSION: KC561137 in the NCBI database, with an overage of 99.78% and identity of 100%. Plasmid Col3M_1 is highly consistent with ACCESSION: JX514065, with an overage of 100% and identity of 98.09%.

### Phylogenetic analysis

**Fig. 2:**
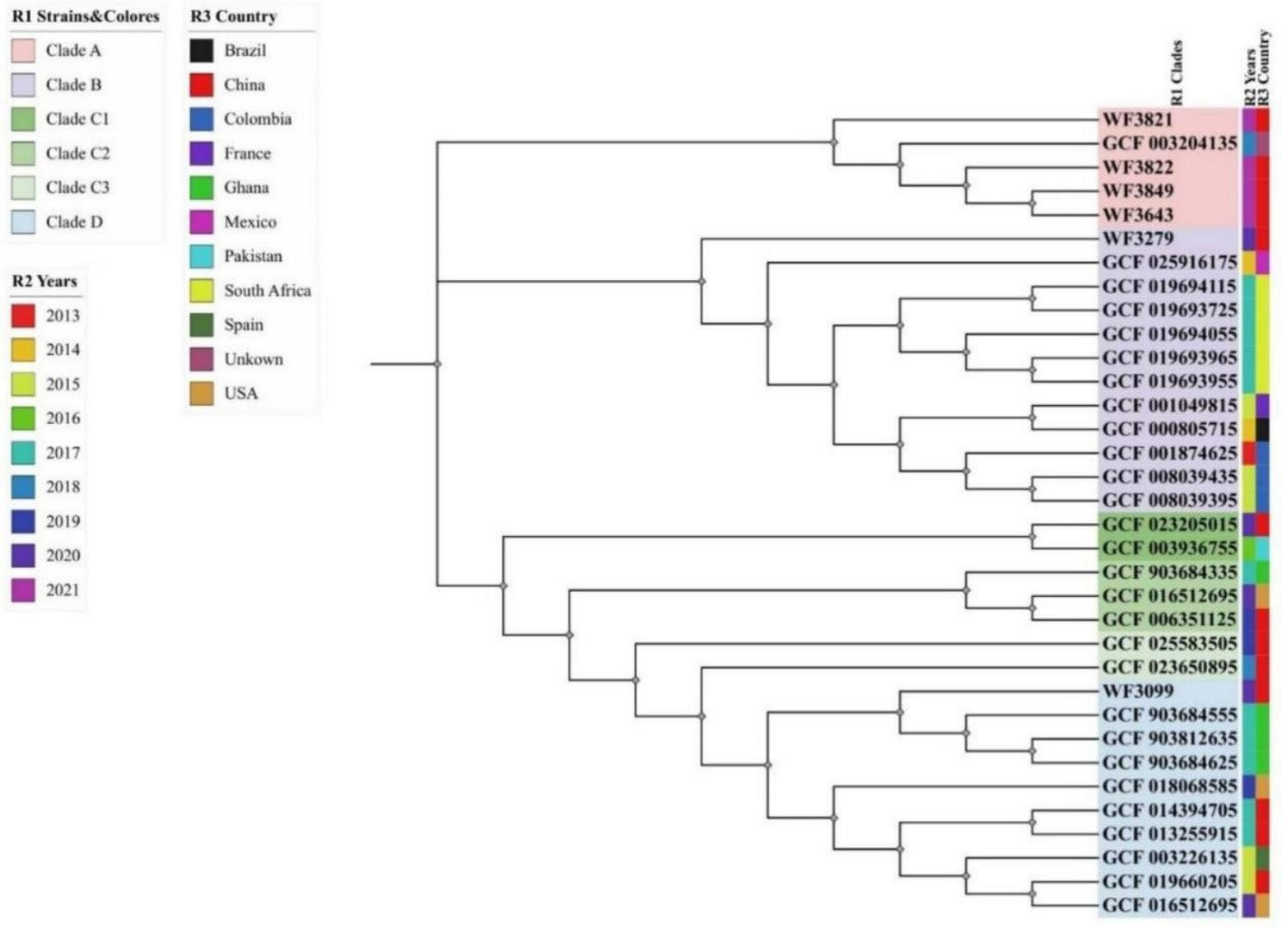
Phylogenetic tree of six carbapenem-resistant *P. rettgeri* isolated in this study and another 28 *P. rettgeri* isolates in NCBI database

The six *P. rettgeri* strains isolated in this study were used to construct a phylogenetic tree together with the 28 *bla*_NDM−1_-carrying *P. rettgeri* strains from the NCBI database. The results of this analysis (Fig. 2) showed that the 34 *P. rettgeri* strains were divided into four clusters, in which the six *P. rettgeri* strains detected in this study were in clade A, clade B, or clade D. The WF3643, WF3849, WF3822, and WF3821 strains are on the same branch and belong to clade A. These strains are closely related, and they are highly homologous to the *P. rettgeri* strain isolated in 2018. The WF3279 strain is in clade B and highly homologous to the *P. rettgeri* strain isolated in Mexico in 2014. The WF3099 strain is in clade D and homologous to the strain isolated in Ghana in 2017. Three strains were highly homozygous, whereas the WF3099 strain was most distantly related to the other five strains. All six *P. rettgeri* strains obtained in this study were highly homologous to the 2017–2018 isolates in the NCBI database as well as to those from South Africa, Ghana, and Mexico.

## Discussion

*Providencia* spp. are normal flora of the human and animal intestines and are important conditional pathogens of medical-acquired infections, with urinary tract and respiratory tract infections being more common [12]. Furthermore, these bacterial species are widely distributed in the natural and hospital environment and have a high likelihood of dissemination [12, 13]. The incidence of *Providencia* spp. infections has increased in recent years, with these infections being associated with widespread sites of infection, infection recurrence, poor prognosis, and even nosocomial outbreaks [14].

In this study, we showed that the *P. rettgeri* strains isolated from the clinical specimens were associated with infections occurring in the urinary tract, respiratory tract, wounds, skin, blood, and other parts of the body, with most strains being isolated from ICUs and surgery wards. Given that these infected patients are middle-aged and elderly people with a history of severe trauma, tumors, hypertension, and various other underlying diseases, and invasive procedures, these conditions may put patients at higher risk of *P. rettger* infection. This finding confirms the results of previous studies [15].

*Providencia* spp. are naturally resistant to several antimicrobial drugs including polymyxins and tigecycline, which poses a serious threat to anti-infective therapy. [16, 17]. Our study results showed that *P. rettgeri* demonstrated high resistance to antibiotics such as β-lactams, aminoglycosides, and sulfonamides, with all six strains showing resistance to SAM, CAZ, CIP, LVX, and ETP and low resistance to FEP, ATM and AMK. Similar to the results of previous study [2, 13], we found that the *Providencia* genus displayed a high degree of resistance to β-lactam antimicrobial drugs, which presents a challenge for the clinical treatment of *P. rettgeri* infections.

In recent years, some strains were found to harbor carbapenem resistance genes, such as *bla*_KPC−2_, *bla*_OXA−48_, *bla*_NDM−1_, *bla*_VIM−2_, *bla*_IMP−27_, and *bla*_IMP−70_, as well as simultaneously carry several other antimicrobial drug resistance genes [18–20], wherein most carbapenemase-encoding genes were shown to be located on plasmids, transposons, or other transposable elements. Most carbapenemase-encoding genes are located on mobile genetic elements (including plasmids and transposons), which can be transmitted between different bacterial species [12, 21], thereby leading to the emergence of carbapenem-resistant strains. In this study, all six strains carried four β-lactam resistance genes (*bla*_OXA−1_, *bla*_OXA−10_, *bla*_VEB−1_, and *bla*_NDM−1_). Most importantly, all six strains also possessed the carbapenemase resistance gene *bla*_NDM−1_, with the presence of carbapenem resistance genes being a major factor mediating carbapenem resistance. This study showed that this bacterium was resistant to both CIP and LVX, and its resistance mechanism may be related to its expression of the quinolone antimicrobial resistance genes *qnrA* and *qnrD*. All *P. rettgeri* strains harboring *bla*_NDM−1_ showed resistance to carbapenem antibiotics, which was consistent with the resistance phenotype. The resistance genotypes and phenotypes of *P. rettgeri* were the same. Three strains of *P. rettgeri* carried aminoglycoside resistance genes but did not show resistance to aminoglycoside antibacterial drugs. However, further investigation is required to determine whether this effect is clinically observed after in vivo administration and to understand the specific mechanism of this phenomenon. Moreover, two strains showed sensitivity to SXT although they carried the sulfonamide resistance gene. The presence of discordance between resistance phenotypes and genotypes in certain strains implies the possibility of distinct resistance mechanisms, thereby necessitating further exploration.

The WF3279 strain carried two plasmids (repUS18_4_rep(pSA8589) and Col3M_1), while three strains (WF3643, WF3822, and WF3849) possessed one plasmid (Col3M_1). The PlasmidFinder predicted that plasmid Col3M_1 was highly similar to p3M-2 A (JX514065) found in *P. vulgaris* in China [22]. Plasmid repUS18_4_rep(pSA8589) was similar to pSA8589 (KC561137) [23]. pSA8589 allows the horizontal transfer of drug resistance genes between different staphylococci [23]. However, our study was based on short-read sequencing, which did not allow us to determine whether the resistance genes existed on the plasmid. Thus, we will conduct further studies to investigate the presence of a horizontal transfer mechanism of the resistance genes.

The homology analysis showed that four strains of *P. rettgeri* were closely related, whereas only one strain isolated from the ICU ward in 2020 was distantly related. The WF3643, WF3849, WF3822, and WF3821 strains of *P. rettgeri* isolated in 2021 were on the same evolutionary branch. Furthermore, these four *P. rettgeri* strains were obtained from different departments, suggesting that their close affinities may be due to the homologous transmission of carbapenem-resistant *P. rettgeri* between some wards in our hospital, which should attract clinical attention. Moreover, bacteria can cross-infect inpatients through medical devices and healthcare workers’ hands, leading to nosocomial outbreaks [5, 20, 24]. Therefore, we should strengthen the surveillance of carbapenem-resistant *P. rettgeri* homology in our hospital to prevent nosocomial infections.

In conclusion, despite the infrequent incidence of human infection by *P. rettgeri*, these infections should be given sufficient attention because they involve a wide range of sites, exhibit high drug resistance, and mostly occur in immunocompromised middle-aged and older patients. Furthermore, the reasonable use of antimicrobial drugs for early treatment, combined with the identification of the infection site and antimicrobial sensitivity testing is necessary to avoid recurrent infections and actively treat the underlying disease. Our study showed that carbapenem-resistant *P. rettgeri* bacteria had caused nosocomial infections in some departments in a small area of our hospital. This finding should attract the attention of clinicians and microbiologists towards the need to strengthen drug resistance monitoring and avoid the generation and spread of pan-drug resistant strains.

### Electronic supplementary material

Below is the link to the electronic supplementary material.

Supplementary Material 1

Supplementary Material 2

## Data Availability

The sequences of the six carbapenem-resistant *P. rettgeri* strains were submitted to GenBank under BioProject PRJNA954298.
