# Supplementary material for: Analysis of resistance genes of carbapenem-resistant Providencia rettgeri using whole genome sequencing
Source: BMC Microbiol. 2023 Oct 3;23:283. doi: 10.1186/s12866-023-03032-3 (PMC10546784; doi:10.1186/s12866-023-03032-3)
Supplement: Supplementary file 1 — Supplementary Material 1 [file 12866_2023_3032_MOESM1_ESM.docx]

**Table S1：**The information of 28 *bla*_NDM-1_-carrying *P. rettgeri* strains from the NCBI database

| Strain | Country | Date |
| --- | --- | --- |
| GCF_000805715 | Brazil | 2014 |
| GCF_006351125 | China | 2019 |
| GCF_013255915 | China | 2017 |
| GCF_014394705 | China | 2017 |
| GCF_019660205 | China | 2015 |
| GCF_023205015 | China | 2020 |
| GCF_023650895 | China | 2018 |
| GCF_025583505 | China | 2019 |
| GCF_005155965 | China | 2017 |
| GCF_001874625 | Colombia | 2013 |
| GCF_008039395 | Colombia | 2015 |
| GCF_008039435 | Colombia | 2015 |
| GCF_001049815 | France | 2015 |
| GCF_903684335 | Ghana | 2017 |
| GCF_903684555 | Ghana | 2017 |
| GCF_903684625 | Ghana | 2017 |
| GCF_903812635 | Ghana | 2017 |
| GCF_025916175 | Mexico | 2014 |
| GCF_003936755 | Pakistan | 2016 |
| GCF_019693725 | South Africa | 2017 |
| GCF_019693955 | South Africa | 2017 |
| GCF_019693965 | South Africa | 2015 |
| GCF_019694055 | South Africa | 2017 |
| GCF_019694115 | South Africa | 2017 |
| GCF_003226135 | Spain | 2018 |
| GCF_003204135 | Unkown | 2015 |
| GCF_018068585 | USA | 2019 |
| GCF_016512695 | USA | 2020 |
