# Supplementary material for: Analysis of resistance genes of carbapenem-resistant Providencia rettgeri using whole genome sequencing
Source: BMC Microbiol. 2023 Oct 3;23:283. doi: 10.1186/s12866-023-03032-3 (PMC10546784; doi:10.1186/s12866-023-03032-3)
Supplement: Supplementary file 2 — Supplementary Material 2 [file 12866_2023_3032_MOESM2_ESM.docx]

**Table S2:**Characteristics of the carbapenem-resistant *P. rettgeri* isolates from the six inpatients

|  | WF3099 | WF3279 | WF3643 | WF3821 | WF3822 | WF3849 |
| --- | --- | --- | --- | --- | --- | --- |
| Gender | Male | Female | Male | Male | Female | Male |
| Age | 21 | 50 | 68 | 48 | 69 | 50 |
| Department | ICU | Cardiology department | Urology Surgery | dermatological department | Maxillofacial Surgery | ICU |
| Diagnosis | Pelvic fracture, pubic bone fracture, sacral fracture | Hypertension, diabetes mellitus, skin ulcers on both lower extremities | Malignant bladder tumor | Skin ulcers | Malignant tumor of tongue | Cranial Trauma |
| Specimen types | Blood | Secretion | Urine | Secretion | Secretion | Sputum |
| isolates date | 20200409 | 20200916 | 20210402 | 20210715 | 20210730 | 20210809 |
| Outcome | Good | General | Good | Poor | Good | Death |
